# Continuous User Experience Monitoring of a Patient-Completed Preoperative Assessment System in the United Kingdom: Cross-sectional Study

**DOI:** 10.2196/31679

**Published:** 2022-01-06

**Authors:** Inocencio Daniel Maramba, Arunangsu Chatterjee

**Affiliations:** 1 Centre for Health Technology University of Plymouth Plymouth United Kingdom

**Keywords:** preoperative assessment, self-completed patient questionnaires, digital health, usability, user experience, web-based

## Abstract

**Background:**

Anesthetic preoperative assessment (POA) is now a common part of the surgical care pathway, and guidelines support its routine use. MyPreOp (Ultramed Ltd) is a web-based POA system that enables remote assessments. Usability is a key factor in the success of digital health solutions.

**Objective:**

This study aims to assess the usability of the MyPreOp system through patient feedback, investigate the amount of time it took for patients to complete the POA questionnaire and the factors that influenced completion time, and explore the effect on completion times of implementing a validated eHealth usability scale, as compared to using a simple but unvalidated usability evaluation scale, and to test the feasibility of administering a more detailed usability evaluation scale in a staggered manner so as not to unduly increase completion times.

**Methods:**

In this cross-sectional study, anonymized data sets were extracted from the MyPreOp system. The participants were adults (aged ≥18 years), scheduled for nonurgent surgical procedures performed in hospitals in the United Kingdom, who gave consent for their anonymized data to be analyzed. Data collected included age, gender, American Society of Anesthesiology (ASA) physical classification status, and completion time. Two user experience evaluations were used: in Phase 1, 2 questions asking about overall experience and ease of use, and in Phase 2, a previously validated usability questionnaire, with its 20 questions equally distributed among 5 succeeding patient cohorts. There were 2593 respondents in total (Phase 1: n=1193; Phase 2: n=1400). The median age of the participants was 46 years, and 1520 (58.62%) of the 2593 respondents were female. End points measured were the median completion times in Phase I and Phase II. The data were collected by extracting a subset of records from the database and exported to a spreadsheet for analysis (Excel, Microsoft Corporation). The data were analyzed for differences in completion times between Phase I and Phase II, as well as for differences between age groups, genders, and ASA classifications.

**Results:**

MyPreOp scored well in usability in both phases. In Phase 1, 81.64% (974/1193) of respondents had a good or better experience, and 93.8% (1119/1193) found it easy to use. The usability rating in Phase 2 was 4.13 out of a maximum of 5, indicating high usability. The median completion time was 40.4 minutes. The implementation of the longer usability evaluation scale in Phase 2 did not negatively impact the completion times. Age and ASA physical status were found to be moderately associated with increased completion times.

**Conclusions:**

MyPreOp rates high in both user experience and usability. The method of dividing the questionnaire into 5 blocks is valid and does not negatively affect completion times. Further research into the factors affecting completion time is recommended.

## Introduction

### Background

Anesthetic preoperative assessment (POA) is now a common part of the surgical care pathway, and guidelines support its routine use worldwide [1]. POA reduces the risk of poor perioperative outcome and reduces cost of a specific group of perioperative candidates [2]. The American Society of Anesthesiologists (ASA) physical status classification score is used worldwide as a comprehensible and practical tool for classifying a patient’s clinical preoperative state, and it correlates well with postoperative mortality [3]. Studies have shown that if age and ASA physical status are known before the preoperative assessment consultation, appointment times can be allocated more accurately [4]. According to the Royal College of Anaesthetists, electronic systems should be considered to enable the capture and sharing of information, support risk identification, and allow data to be collected and made available for audit and research purposes [5]. The implementation of a preoperative digital tool may help to improve guideline adherence [6].

To realize the benefits previously enumerated, a few computerized preoperative assessment systems have recently been developed [2,3,7,8]. One such system that is gaining adoption is MyPreOp.

**Figure 1:**
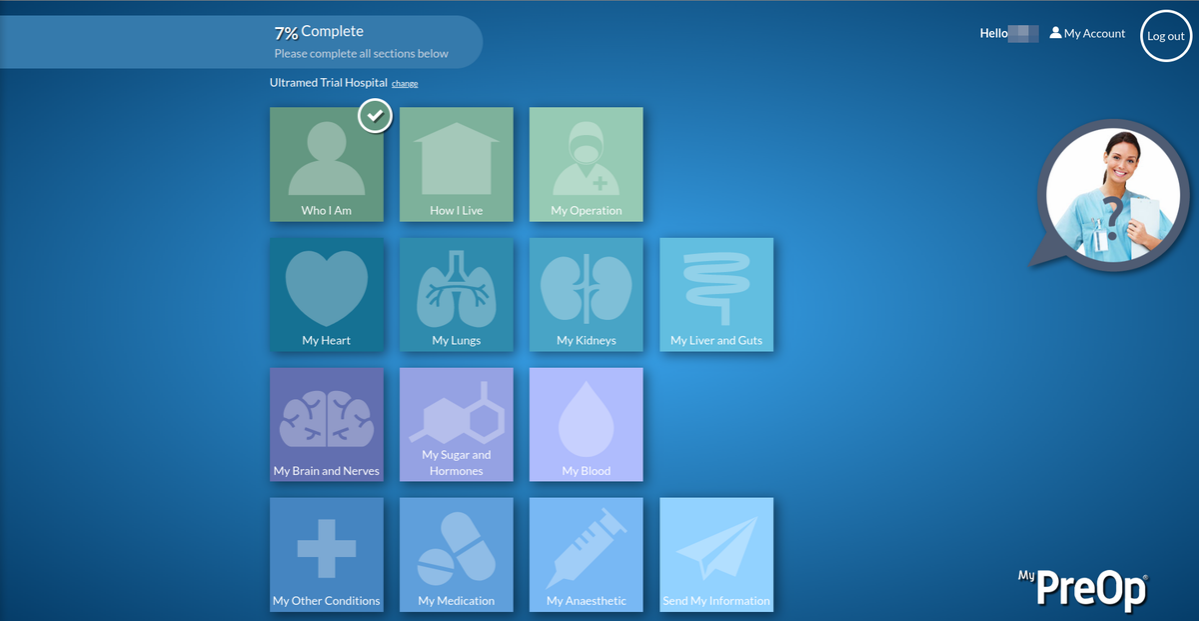
Screenshot of the MyPreOp question page.

MyPreOp (Ultramed Ltd) is a web-based patient facing app using a secure encrypted connection, designed to replace paper-based preoperative assessments. Patients requiring an operation can create an account and complete a comprehensive assessment of their general health and medical history. The output includes a clinical summary providing an ASA risk grade of 1 to 5 and recommends additional tests the patient may need. This information is then submitted via email to a nurse from the preoperative team in the form of a PDF file; the nurse reviews the summary and acts on any information provided. The PDF file can be uploaded to the clinical system. The cloud-hosted service can be accessed using a smartphone, tablet, or home computer. As of January 2020, more than 20,000 patients had used the system across 8 UK hospitals, and this number is increasing as more hospitals adopt the system. Incremental improvements have been made to the system based upon feedback from patients and clinicians. Feedback from patients is sought using a short questionnaire after preoperative details are completed. The data from the MyPreOp system is stored in an SQL relational database hosted on a Microsoft Azure server [9]. A screenshot of one of the question pages of MyPreOp is shown in Figure 1.

Usability has been identified as a key component of good practice in the development of digital applications and is a key criterion for the assessment of digital applications in health. Evaluation of usability in eHealth applications has enormous value for patient benefit, as well as greater acceptance by patients and clinicians alike. It is also necessary to ensure that health technologies are appropriately designed and targeted to the end users’ needs before they are used as health interventions [10-12]. A recent World Health Organization report stressed that development of digital solutions should be focused on user experience [13].

### Study Rationale

We conceived this study to address the need to evaluate a POA system using a validated digital health usability measure. We reviewed the literature to find similar studies that evaluated the usability of a POA system.

In the literature, we found only two computerized preoperative assessment systems that reported the results of the usability testing methods used in their development and evaluation. The developers of one system used the System Usability Scale (SUS) questionnaire as well as a heuristic evaluation to evaluate usability [7]. The SUS is a commonly used usability evaluation scale, but it is not specific to digital health applications.

User experience and acceptability of another system were measured using the Questionnaire’s Questionnaire, 10-item version (QQ-10) [1]. The QQ-10 was developed and validated to evaluate specific aspects of value and burden of a questionnaire. However, it was originally intended for paper questionnaires, and it was not specifically designed to evaluate the usability of digital health applications.

Current evidence is limited on the factors influencing the amount of time it takes for patients to self-complete a computerized preoperative assessment. A previous study has shown that age and ASA physical status have the largest effect sizes in influencing the amount of time spent in a face-to-face preoperative assessment with a clinician [4]. We decided to investigate the patient-reported usability of the MyPreOp preoperative assessment system with a validated digital health usability measure, assess the factors that influence the completion times, and devise a method of continuously assessing usability without unduly increasing completion time.

### Study Aims and Objectives

The aim of our study was to evaluate the usability of the MyPreOp preoperative assessment system. The objectives of the study were to assess the usability of the system through patient feedback, investigate the amount of time it took for patients to complete the preoperative assessment questionnaire and the factors that influence completion time, and explore the effect on the completion times of implementing a validated eHealth usability scale as compared to using a simple but unvalidated usability evaluation scale, and test the feasibility of administering a more detailed usability evaluation scale in a staggered manner so as not to unduly increase completion times.

## Methods

### Study Design, Setting, and Recruitment

The research was designed as a cross-sectional study, with all participants selected based on the inclusion and exclusion criteria for the study. The inclusion criteria were patients aged 18 years and older who entered data in the MyPreOp system for preoperative assessment for nonurgent surgical procedures in hospitals across the United Kingdom and successfully completed the POA.

### Intervention and Data Collection (Phase 1 and Phase 2)

The study was performed in two phases. An initial analysis of retrospectively extracted data was performed for Phase 1 of the study. In Phase 2, data were prospectively collected, and a longer, previously validated usability questionnaire was used to evaluate the usability of the system.

#### Phase 1

For the first phase of the study, an anonymized subset of the data was extracted from records entered by patients into the MyPreOp database from January to March 2019 and exported to a spreadsheet (Excel 2013, Microsoft Corporation). The variables in the subset included age, gender, ASA grade, and amount of time required to complete the questionnaire (completion time). Completion time was measured by measuring the time from when the patient logged on to the system to when the patient clicked on the submission button. Data from the responses to two feedback questions about their overall experience and ease of use of the system were also extracted. The two questions were:

- Question 1: “Overall, how did you find your experience of using MyPreOp?” Respondents were asked to choose between Excellent, Good, Satisfactory, and Poor.
- Question 2: “How easy did you find it to follow the instructions and enter your information?” The choices were Very easy, Easy enough, A bit difficult, and Very difficult.

A free text comment box was also included for patients to enter any comments or suggestions.

#### Phase 2

In Phase 2, data were extracted from patient entries made in the period from the start of May 2019 to mid-June 2019. Age, gender, ASA grade, and the amount of time needed to complete the MyPreOp questionnaire were among the variables collected.

The patients evaluated the usability of the system by filling in the Health Information Technology Usability Evaluation Scale (Health-ITUES). The Health-ITUES is a validated instrument that explicitly considers each task by addressing various levels of expectation of support for the task by the health information technology. The Health-ITUES also has the added benefit of being customizable; it can address the study needs and concepts measured without item addition, deletion, or modification. The Health-ITUES has been validated in both web and mobile health technologies. The Health-ITUES consists of 20 items rated on a 5-point Likert scale from strongly disagree (1) to strongly agree (5). A higher scale value indicates higher perceived usability of the technology [14,15].

### End Points Measured

The main outcome of interest was the time taken to complete the preoperative assessment. Explanatory variables were age, gender, and ASA physical classification, as previous studies had identified these as variables that were correlated with differences in completion times in face-to-face preoperative assessments [4].

### Study of the Intervention

To lessen the effect from “questionnaire fatigue,” the 20 questions of the Health-ITUES were divided into 5 blocks of 4 questions each. Each block of questions was presented to the users of MyPreOp for approximately 1 week before switching to the next block of questions.

It was hypothesized that if the patients had a clearer idea of how long it would take to complete the assessment, they would be able to manage their expectations better and this would increase their perception of the system’s usability. During Phase 2, patients using the system in the first 2 weeks of the month used the usual system, and in the latter 2 weeks, this message was included at the start of the assessment:

Most people take between 30 and 60 minutes to complete MyPreOp. This is because your hospital needs a lot of background information about you. I understand that it is likely to take me between 30 and 60 minutes to complete MyPreOp.

The completion times and ratings of patients who did not see the message and patients who saw the message were compared.

### Data Analysis

The data analysis was organized as follows:

- Assessment of usability: the usability of MyPreOp was assessed through ease-of-use questions and by the Health-ITUES score.
- Questionnaire completion times and the factors affecting this (age, gender, ASA classification): we analyzed the effects of these factors on average completion times, as well as the correlation between completion times and age.
- Effect of using a more detailed usability scale (Health-ITUES): we also looked at the correlation between completion times and usability as measured by the Health-ITUES.

Analysis of the data consisted of computation of descriptive statistics and tests of the differences between completion times and ages. Preliminary analyses were conducted to ensure that there was no violation of the assumptions of each statistical test. Depending on the nature of the data, an appropriate statistical test was used to test the differences between the continuous variables under investigation. If the data were normally distributed, then a *t* test (for 2 groups) or analysis of variance (for more than 2 groups) was used. In cases where the data were not normally distributed, the Wilcoxon rank sum test (2 groups) or Kruskal-Wallis test (more than 2 groups) was used where appropriate. A *P* value <.05 was considered statistically significant. For categorical variables, the chi-square test was used to test the differences in proportions. All statistical analyses were performed using the R statistical package (R Foundation for Statistical Computing).

### Ethical Considerations

Anonymized data sets were provided to the investigators by the service provider (Ultramed). The study was classified as a service evaluation, which, together with the use of anonymized data that had been collected by a second party with informed consent, meant that formal ethical approval was not necessary.

We also accomplished the mHealth Evidence Reporting and Assessment (mERA) checklist, which is included as Multimedia Appendix 1.

## Results

### Participant Characteristics

In Phase 1, data from 1236 patient entries into the MyPreOp system were collected, with complete data available for 1193 patients. In Phase 2, data from 1496 patient entries were collected, with complete data available for 1400 patients. Complete data were available for 2593 patients in total. The baseline characteristics, including age, gender, time to complete MyPreOp, and responses to the feedback questions on overall experience and ease of use are shown in Table 1. In terms of age, participants in Phase 1 were younger than participants in Phase 2, and the difference was statistically significant (Wilcoxon rank sum test, *P*<.001). Likewise, the completion times for patients in Phase 1 were shorter than the completion times for patients in Phase 2, and the difference was statistically significant (Wilcoxon rank sum test, *P*<.001). On average, Phase 1 patients completed the MyPreOp assessment 6.97 (95% CI 7.53-4.66) minutes faster than Phase 2 patients.

The median completion times of the different age groups and ASA physical status groups varied significantly from one another (Table 2). Overall, there was no difference in median completion times between male and female participants (Wilcoxon rank sum test, *P*=.28). The median completion times for each ASA physical status group were statistically different from those of each of the other ASA physical status groups (*P*<.001). This was true for both phases.

There was a statistically significant difference in the median ages of each of the ASA physical status groups (Kruskal-Wallis test, *P*<.001). A pairwise comparison showed that this difference is statistically significant between the ASA physical status groups, except for between ASA grades 3 and 4. The table below shows the median ages for the ASA physical status groups.

This indicates that age and ASA grade are not independent of each other and that as age increases, the ASA grade likewise increases.

**Table 1:** Summary of demographics, ASA status, and time to complete MyPreOp.

Characteristic			Overall (N=2593)^a^		Phase 1 (n=1193)^b^		Phase 2 (n=1400)^c^	
			n (%)	Median time to complete MyPreOp (minutes)	n (%)	Median time to complete MyPreOp (minutes)	n (%)	Median time to complete MyPreOp (minutes)
**Age (years)**								
	≤45	1290 (49.75)		32.16	706 (59.18)	30.79	584 (41.72)	33.84
	46-65	799 (30.81)		43.28	334 (27.30)	40.78	465 (33.21)	44.48
	≥66	504 (19.44)		61.17	153 (12.82)	59.68	351 (25.07)	61.85
**Gender**								
	Female	1520 (58.62)		40.7	698 (58.51)	38.32	822 (58.71)	43.32
	Male	1073 (41.38)		40.12	495 (41.49)	34.5	578 (41.29)	44.57
**American Society of Anesthesiology physical status**								
	1	953 (36.75)		32.48	499 (41.83)	31.23	454 (32.43)	34.54
	2	1021 (39.38)		42.6	434 (36.38)	39.36	587 (41.93)	45.15
	3	526 (20.29)		51.35	220 (18.44)	45.14	306 (21.86)	55.04
	4	93 (3.58)		60.55	40 (3.35)	54.62	53 (3.78)	63.57

^a^Median age 46 years; median time to complete MyPreOp 40.47 minutes.

^b^Median age 40.88 years; median time to complete MyPreOp 36.75 minutes.

^c^Median age 50.96 years; median time to complete MyPreOp 43.92 minutes.

**Table 2:** Median ages of the American Society of Anesthesiology (ASA) groups.

ASA physical status group	Age (years), median
1	35
2	49
3	59
4	63

### Assessment of Usability: Phase 1

In Phase 1, complete results were obtained from 1193 patients. For the evaluation, patients were asked two multiple choice questions about overall experience and ease of use, as stated in the *Methods* section of this paper. The frequency of the responses to the two evaluation questions, median time, and ASA physical status for each type of response are shown in Table 3.

**Table 3:** Patient satisfaction ratings, completion times, ASA physical status, and overall experience in Phase 1.

Phase 1 (n=1193)		Overall experience				Ease of use			
		Excellent	Good	Satisfactory	Poor	Very easy	Easy enough	A bit difficult	Very difficult
Participants, n (%)		417 (34.95)	557 (46.69)	194 (16.26)	25 (2.10)	697 (58.43)	422 (35.37)	63 (5.28)	11 (0.92)
Median time to complete MyPreOp (minutes)		32.67	38.33	44.17	42.33	33.18	41.94	53.13	35.05
**American Society of Anesthesiology physical status**									
	1	200 (16.76)	222 (18.60)	69 (5.78)	8 (0.67)	331 (27.75)	149 (12.49)	17 (1.42)	2 (0.17)
	2	149 (12.49)	206 (17.27)	74 (6.20)	5 (0.42)	244 (20.45)	157 (13.16)	28 (2.35)	5 (0.42)
	3	59 (4.95)	110 (9.22)	41 (3.44)	10 (0.84)	107 (8.97)	98 (8.21)	11 (0.92)	4 (0.34)
	4	9 (0.75)	19 (1.59)	10 (0.84)	2 (0.17)	15 (1.26)	18 (1.50)	7 (0.59)	0 (0.00)

Median completion times differed statistically depending on the response given in the overall experience question (*P*<.001). Those who answered that they had an “excellent” overall experience had a quicker median completion time (32.67) than those who answered “poor” to the overall experience question (42.33). Those who answered that they found MyPreOp “very easy” to use had a quicker media completion time of 33.18 minutes; this was slightly faster than those who found the system “very difficult” to use, with a median completion time of 35.05 minutes.

The responses to the feedback questions were related to the ASA physical status scores computed by the MyPreOp system.

A chi-square test revealed that there was a significant association between ASA physical status and the response to the overall experience feedback question (*χ*^2^_9_=25.793; *P*<.05).

Likewise, the responses to the ease-of-use question were also significantly associated with ASA physical status. The chi-square test showed that the association between ASA physical status and the response to the ease-of-use question was significant (*χ*^2^_9_=42.75; *P*<.001).

### Assessment of Usability in Phase 2: Health Information Technology Usability Evaluation Scores

In Phase 2, complete entries were collected from 1400 patients. As in Phase 1, data on age, gender, computed ASA physical status classification, and number of minutes needed to complete the assessment were collected. In addition to this, the simple evaluation questions in Phase 1 were replaced with more detailed questions from the Health-ITUES. As described previously, the 20 questions in the Health-ITUES were divided into 5 blocks of 4 questions each and were presented sequentially over the data collection period. Each patient answered 4 of the 20 questions. The questions are divided into the following domains: impact, perceived usefulness, perceived ease of use, and user control. The median score for each question, as well as the median combined and overall scores, are shown in Table 4.

**Table 4:** Descriptive statistics for the question scores.

Question		Median score	Median combined score
**Impact**			4.09
	I think MyPreOp would be a positive option for persons needing to fill out a preoperative assessment.	4.11	
	I think MyPreOp would improve the quality of care of persons needing to complete a preoperative assessment.	4.03	
	MyPreOp is an important part of meeting my needs related to preoperative assessment and my upcoming operation.	4.11	
**Perceived usefulness**			4.19
	I am able to self-complete my preoperative assessment in a timely manner because of MyPreOp.	4.12	
	Using MyPreOp is useful for self-completion of my preoperative assessment.	4.13	
	I think MyPreOp presents a modern approach for the self-completion of preoperative assessment.	4.43	
	Using MyPreOp makes it easier to self-complete my preoperative assessment.	4.38	
	Using MyPreOp enables me to self-complete my preoperative assessment more quickly.	4.1	
	I am able to make changes to my medical history myself whenever I use MyPreOp.	4.05	
	Using MyPreOp increases my ability to self-complete my preoperative assessment.	4.2	
	I am satisfied with MyPreOp for self-completion of my preoperative assessment.	4.16	
	Using MyPreOp makes it more likely that I can self-complete my preoperative assessment.	4.11	
**Perceived ease of use**			4.26
	I can always remember how to log on and use MyPreOp.	4.21	
	I find MyPreOp easy to use.	4.19	
	Learning to operate MyPreOp is easy for me.	4.37	
	I am comfortable with my ability to use MyPreOp.	4.38	
	It is easy for me to become skillful at using MyPreOp.	4.16	
**User control**			4.24
	MyPreOp gives error messages that clearly tell me how to fix problems.	4.4	
	Whenever I make a mistake using MyPreOp, I can amend answers easily and quickly.	4.23	
	The information (such as online help, on-screen messages, and other documentation) provided with MyPreOp is clear.	4.05	
Overall score		N/A^a^	4.2

^a^N/A: not applicable.

To determine if the scores could be combined into scales, the median scores for each question block were calculated and compared using the Kruskal-Wallis test. This nonparametric test was chosen because the data were not normally distributed, as demonstrated by a Shapiro-Wilk test. The Kruskal-Wallis test showed that the differences between the median scores of the blocks were statistically significant (*P*<.001). The effect size was calculated to determine the extent of the differences of the scores between blocks using the *ε*^2^ statistic. The calculated *ε*^2^ value was 0.011, which is interpreted as indicating a small effect size [16]. Furthermore, the effect size of the differences in the scores of each question was calculated. The calculated *ε*^2^ value was 0.02, which also corresponds to a small effect size. We therefore determined that combining the scores was a valid approach because the effect size of dividing the questionnaire into blocks was minimal.

MyPreOp scored well in all the subscales and had a median overall score of 4.2 out of a maximum score of 5 in usability as measured by the Health-ITUES. The subscale where MyPreOp scored highest was perceived ease of use (4.26/5), and the subscale with the lowest score was impact (4.09/5).

We investigated if the ratings differed between age categories. The results are shown in Table 5.

The youngest age category gave the highest median scores (4.37), while the oldest age category gave the lowest median ratings (3.94). The differences in ratings were quite small (0.43), and calculating for effect size showed that the age category had only a small effect size on the ratings (effect size 0.04).

**Table 5:** Median overall ratings by age category.

Age category (years)	Median overall score (out of 5)
≤45	4.37
46-65	4.22
≥66	3.94

### Factors Affecting Completion Times

Using the combined data from both phases, the research team investigated which factors influenced the amount of time required for patients to complete the preoperative evaluation. The factors examined included age category, ASA grade, gender, and use of the Health-ITUES (this scale was not used in Phase 1, whereas it was used in Phase 2).

**Figure 2:**
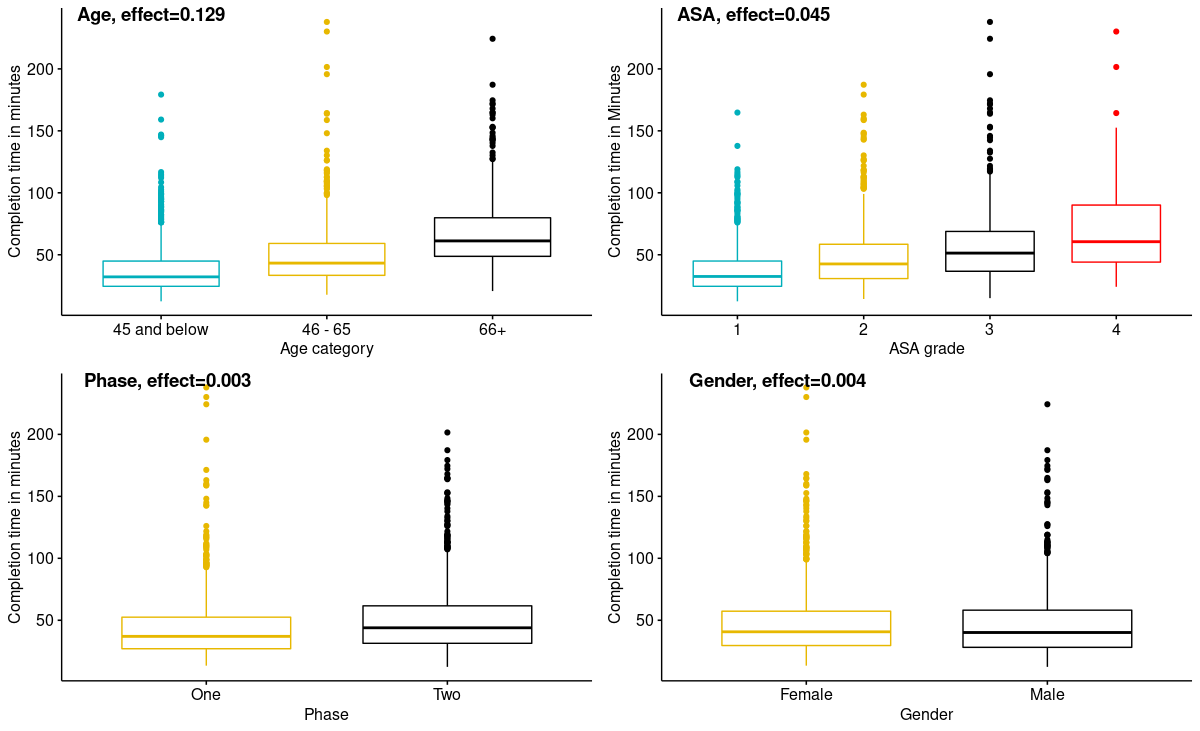
Box plots of the completion times and effect sizes of various factors. ASA: American Society of Anesthesiology.

The box plots in Figure 2 illustrate the completion times for each category of the factors, as well as the effect size for each factor. Comparing the median completion times for the various factors under investigation, we found that the completion time increases with age and that the age category has the largest effect size. Patients aged ≤45 years had a median completion time of 32.16 minutes, compared with 61.17 minutes for patients aged ≥66 years, a difference of 29.01 minutes. Completion time also increased with ASA physical status grade; the median completion time of patients with ASA grade 1 was 28.07 minutes shorter than that of patients with ASA grade 4. Incorporating the Health-ITUES scale in Phase 2 (in 5 blocks of 4 questions each) had only a small effect on completion time, with an increase of 7.17 minutes for the cohort using the Health-ITUES. The computed effect size of including the Health-ITUES was likewise very small, accounting for only 0.3% of the variance. Gender also had a negligible effect on completion time, with a <1 minute difference in completion times between female and male participants.

**Figure 3:**
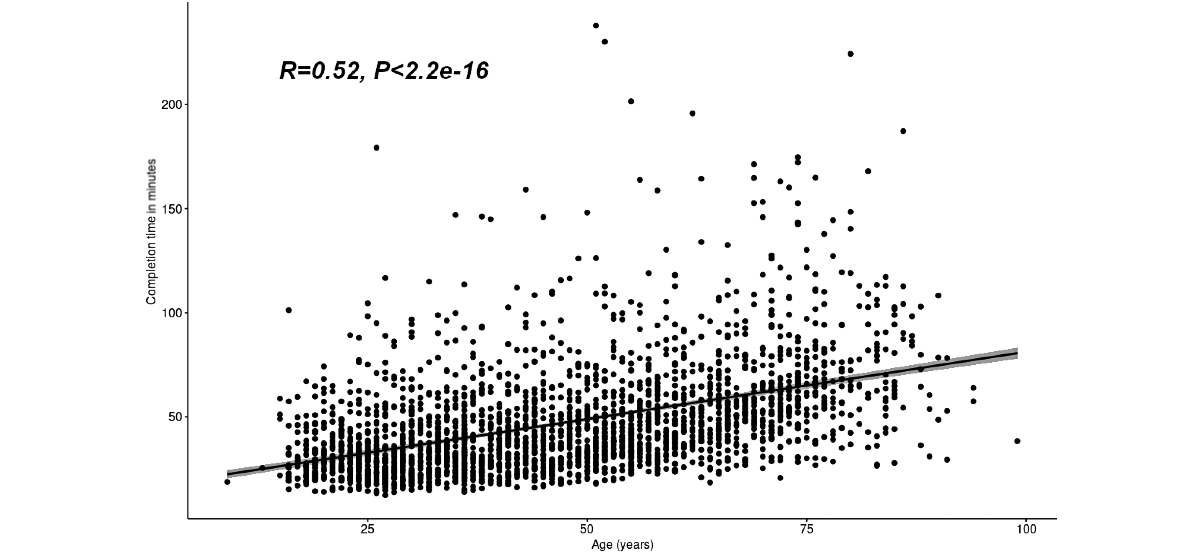
Correlation plot of age and completion time.

Although age had the largest effect size on completion time, it only accounted for approximately 12% of the variance in completion times, as indicated by the partial *ε*^2^ value. We further investigated the correlation between age and completion time by plotting the times on a graph and calculating the correlation. The results are shown in Figure 3. The calculated correlation coefficient was 0.52 (*P*<.001), indicating a moderate positive correlation of age with completion time.

### Effect of Completion Times on Health-ITUES Ratings

**Figure 4:**
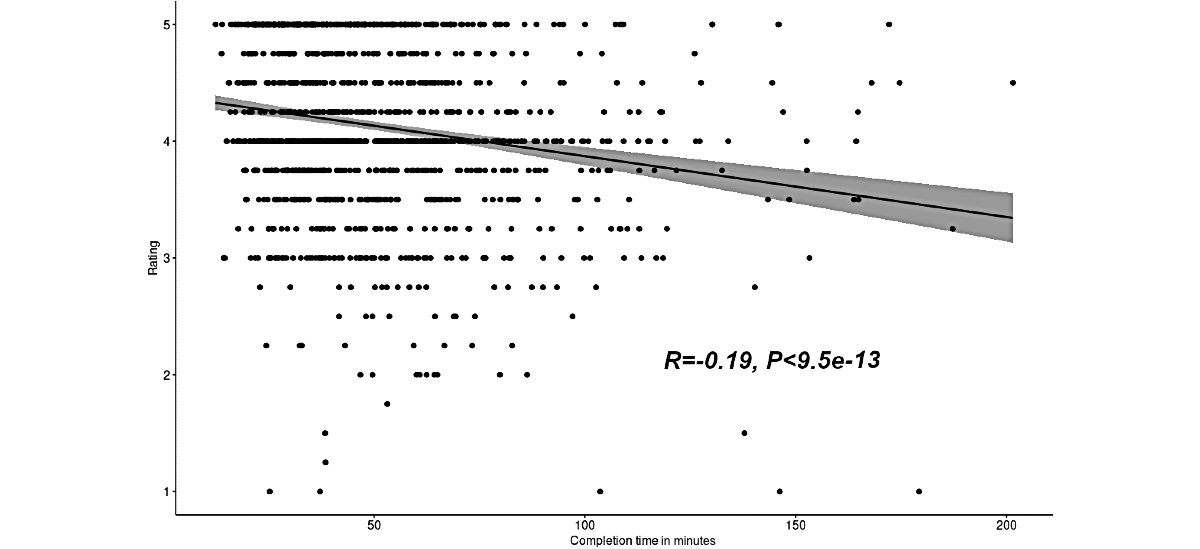
Correlation plot of the completion times and ratings in Phase 2.

We also examined the relationship between completion times and the overall Health ITUES ratings. The ratings in the four questions asked for every patient were averaged to obtain an overall rating for every patient. We then plotted the overall ratings with their corresponding completion times and calculated the correlation coefficient to determine the strength of the association. The results are shown in Figure 4. The calculated correlation was –0.19 (*P*<.001), which is evaluated as a weak negative correlation between the rating and completion time. This can be interpreted as a very slight decrease in the usability rating as the completion time increases.

Finally, the ratings and completion times of patients who received the information message about the time needed to complete the assessment were compared. The median overall ratings by patients who did not receive the message and patients who received the message were both 4. There was no significant difference between the ratings (*P*=.41). The median completion times for the groups who did not receive the message and who received it were 43.6 minutes and 44.3 minutes, respectively. There was no statistical difference between completion times for the 2 groups (Wilcoxon rank sum test, *P*=.50). We interpret this as the appearance of the information message having no effect on either the rating or completion time.

In summary, the outcomes of interest, as in, the completion times and usability ratings, were influenced by contextual factors. Age and ASA physical status had the most effect on completion times, while gender, the use of the Health-ITUES questionnaire, and the presence of a message about the length of time needed to complete the evaluation had little effect on completion times. The presence of the message also had no effect on the usability ratings. Completion time had a weak negative effect on usability ratings. We cannot discount the possibility of other factors influencing the completion time, such as speed and quality of the internet connection, whether the patient took a break while completing the assessment, and whether it was the patient or a carer who completed the assessment. We explore this further in the *Discussion* section.

## Discussion

### Principal Results

The results of both Phase 1 and Phase 2 indicate that MyPreOp scores very high in overall experience, ease of use, and usability as measured by the Health-ITUES evaluation scale. Approximately 81% of the users in Phase 1 rated their overall experience of MyPreOp as excellent or good, and 94% rated the system’s ease of use as very easy or easy enough. Older people and people with more morbidities (as indicated by age category and ASA physical status score) tended to give lower ratings for overall experience and ease of use.

The Health-ITUES ratings collected in Phase 2 show that the participants rated MyPreOp high in usability, with an overall rating of 4.13 out of 5. Age only had a small effect on the ratings, indicating that both younger and older patients perceived the system as highly usable. Completion time also had little effect on the ratings, as the correlation between the ratings and the time taken to complete the assessment was weak. This finding demonstrated that the high perceived usability of MyPreOp was due primarily to the user interface design and was not affected by other factors.

Dividing the 20 questions of the Health-ITUES in 5 blocks of 4 questions had little effect on the variance of the ratings and on the completion times. This shows that it is feasible to administer the Health ITUES in this fashion, provided that the block sizes are large enough. Our study had block sizes ranging from 252 to 311 patients per block, which would provide adequate power.

We interpret the smaller effects that age and other factors such as ASA physical status had on the ratings as being due to the more specific nature of the questions being asked in the Health-ITUES. The benefit of the Health-ITUES is that it is able to provide ratings for specific subscales; this will be useful in designing the next iteration of MyPreOp, with the aim of providing a better user experience. The simple questions about overall experience and perceived ease of use only provide a general idea about what can be improved upon in the user experience. The fact that completion time had little effect on the ratings but a significant effect on overall experience can be attributed to user experience encompassing factors other than usability, such as usefulness, desirability, accessibility, credibility, findability, and value [17].

The results indicate that increased completion times are associated with increasing age and ASA physical status, as in, older patients with more disease conditions take longer to complete the self-assessment. A characteristic of the system is that because of the branching algorithms, those with more morbidities will be asked more questions, thereby increasing completion times. This corresponds to the findings from a study that measured the time of in-clinic POA with health professionals, where age and ASA physical status were also associated with increased length of assessment [4].

Aside from the increased number of morbidities that accompany advancing age, other factors associated with age may be present that increase completion time. It is acknowledged that at present there is a digital divide, where older people are less familiar with digital technology. This may also contribute to the increase in completion times for a web-based self-administered questionnaire in older people with less digital health literacy. Training programs, possibly delivered by digital health champions, may be able to raise digital health literacy in older populations [18-20].

It is important to note that while age had the greatest effect size on completion time among the factors being compared, the actual measured effect size was small, accounting for only 12% of the variance in completion times. This indicates that other factors that were not measured in the data set could be responsible for increasing completion times. These factors could include the nature of the internet connection being used (eg, mobile phone network vs superfast broadband), the device being used (smartphone, tablet, or PC), whether a patient or a carer is answering the assessment. Another factor to be taken into consideration is whether the patient answered the question in one sitting or took breaks or “multitasked” while accomplishing the assessment. The fact that the user can save their data, perform other tasks, and return to the assessment could be one factor that accounts for the high overall experience and usability scores despite the completion times ranging from 12 to 237 minutes. The convenience of being able to complete the assessment when and where the patient chooses, coupled with the ability to save the data and resume the assessment, is one of the desirable features of the system. The system also gives informational links relevant to the disease conditions being reported by the patient. Time spent browsing these links also gets included in the completion time if the patient decides to read the information before completing the assessment.

### Comparison With Prior Studies

Our findings are similar to the study performed by Hawes et al [4], who also found that age and ASA physical status influenced the length of face-to-face preoperative assessment. Some of the other factors that they found to influence assessment time were which nurse practitioner saw the patient and the type of surgery the patients were being screened for. The former factor is not applicable in our study, as all the participants in our study performed self-assessments. Data on the type of surgery were not collected in our study. However, the effect size of the type of surgery was very small, only 0.006, meaning that it accounted for only 0.6% of the variability.

### Study Limitations

The lack of data on the other factors that affect completion time is one of the limitations of the study. Data on the speed of the respondent’s internet connection, the type of device they used to complete the assessment, whether the patient and/or a carer answered the questionnaire, and the digital health literacy of the respondent are not routinely collected by the MyPreOp system. Further studies are needed to collect data on these factors and determine the extent of their influence on completion times. Data on the type of surgery being screened for were also not collected.

The strength of the study lies in the large sample size from which we were able to collect data. To our knowledge, this is the first study of a POA system that used a usability evaluation scale designed specifically for health information technology.

### Conclusion

The objectives of the study were to (1) evaluate the usability of MyPreOp, (2) investigate the factors that influence the time it takes to complete MyPreOp, and (3) explore how a validated usability measure can be implemented without unduly lengthening completion times.

We found that for the majority of the 2593 patients whose data were included in the study, MyPreOp provides a good overall experience, good perceived ease of use, and high usability as measured by both simple usability questions and the Health-ITUES. The factors that influenced completion time were age and ASA physical status. The method of administering the Health-ITUES by administering it 4 questions at a time in 5 blocks did not have a deleterious effect on completion times or induce a large variation in the ratings, as shown by the small effect size.

One lesson we learned was that for digital health applications with a large installed base, dividing the usability questionnaire items into more manageable blocks is a valid way of evaluating user experience without negatively impacting completion times. This prevents “questionnaire fatigue” on the part of the respondents.

More research is needed on the factors that influence completion times, setting aside the inherent nature of the system where more questions are asked when the patient has multiple disease conditions. It would also be helpful to further explore the impact of a message addressing length of assessment completion at the start of the assessment. Additional qualitative research is also needed on what factors specifically impact on user experience. The association between digital health literacy and the speed of completion of electronic health questionnaires needs to be investigated further.

We recommend that training programs aimed at increasing the digital health literacy of the older population be instituted, as this can contribute to faster completion times for computerized preoperative self-assessment as well as other self-completed computerized assessments. We also recommend that digital health champions be deployed to assist in delivering programs to increase digital health literacy in the older population.

## Appendix

Multimedia Appendix 1 mHealth evidence reporting and assessment (mERA) checklist.

## Abbreviations

ASA: American Society of Anesthesiology
Health-ITUES: Health Information Technology Usability Evaluation Scale
POA: preoperative assessment
QQ-10: Questionnaire’s Questionnaire 10-item version
SUS: System Usability Scale
